# What maximizes the effectiveness and implementation of technology-based interventions to support healthcare professional practice? A systematic literature review

**DOI:** 10.1186/s12911-018-0661-3

**Published:** 2018-11-07

**Authors:** C Keyworth, J Hart, C J Armitage, M P Tully

**Affiliations:** 1 Manchester Centre for Health Psychology, Division of Psychology and Mental Health, School of Health Sciences, Faculty of Biology, Medicine and Health, University of Manchester, Manchester Academic Health Science Centre, Coupland 1 Building, Oxford Road, Manchester, M13 9PL UK; 2 Division of Medical Education, School of Medical Sciences, Faculty of Biology, Medicine and Health, University of Manchester, Manchester Academic Health Science Centre, Oxford Road, Manchester, M13 9PL UK; 3 Division of Pharmacy and Optometry, School of Health Sciences, Faculty of Biology, Medicine and Health, University of Manchester, Manchester Academic Health Science Centre, Stopford Building, Oxford Road, Manchester, M13 9PL UK; 4 NIHR Manchester Biomedical Research Centre, Manchester University NHS Foundation Trust, Manchester Academic Health Science Centre, Manchester, M13 9PL UK; 5 NIHR Greater Manchester Patient Safety Translational Research Centre, Manchester, UK

**Keywords:** Healthcare professional behaviour change, Technology, Realist review, Intervention, eHealth

## Abstract

**Background:**

Technological support may be crucial in optimizing healthcare professional practice and improving patient outcomes. A focus on electronic health records has left other technological supports relatively neglected. Additionally, there has been no comparison between different types of technology-based interventions, and the importance of delivery setting on the implementation of technology-based interventions to change professional practice. Consequently, there is a need to synthesise and examine intervention characteristics using a methodology suited to identifying important features of effective interventions, and the barriers and facilitators to implementation. Three aims were addressed: to identify interventions with a technological component that are successful at changing professional practice, to determine *if* and *how* such interventions are theory-based, and to examine barriers and facilitators to successful implementation.

**Methods:**

A literature review informed by realist review methods was conducted involving a systematic search of studies reporting either: (1) behavior change interventions that included technology to support professional practice change; or (2) barriers and facilitators to implementation of technological interventions. Extracted data was quantitative and qualitative, and included setting, target professionals, and use of Behaviour Change Techniques (BCTs). The primary outcome was a change in professional practice. A thematic analysis was conducted on studies reporting barriers and facilitators of implementation.

**Results:**

Sixty-nine studies met the inclusion criteria; 48 (27 randomized controlled trials) reported behavior change interventions and 21 reported practicalities of implementation. The most successful technological intervention was decision support providing healthcare professionals with knowledge and/or person-specific information to assist with patient management. Successful technologies were more likely to operationalise BCTs, particularly “instruction on how to perform the behavior”. Facilitators of implementation included aligning studies with organisational initiatives, ensuring senior peer endorsement, and integration into clinical workload. Barriers included organisational challenges, and design, content and technical issues of technology-based interventions.

**Conclusions:**

Technological interventions must focus on providing decision support for clinical practice using recognized behavior change techniques. Interventions must consider organizational context, clinical workload, and have clearly defined benefits for improving practice and patient outcomes.

**Electronic supplementary material:**

The online version of this article (10.1186/s12911-018-0661-3) contains supplementary material, which is available to authorized users.

## Background

Changing healthcare professional practice is fundamental to the implementation of any health policy, intervention or safety measure intended to deliver best patient care. This is particularly important given the responsibilities that healthcare professionals have with respect to patient management and improving health outcomes [1]. Previously targeted behaviors include prescribing medication [2], conducting screening and health checks [3, 4], providing support and making appropriate referrals [5], and making diagnoses [6]. Optimizing performance of these target behaviors provides an opportunity to influence directly the clinical management of patients and hence accelerate improvement in patient care and/or patient outcomes.

Technology-based interventions can address known barriers in the work environment such as time and workload pressure [7] and provide an opportunity to exert greater impact on patient outcomes by changing professional practice rather than changing the behavior of patients one-by-one. Interventions with a technological component include automated prompts and reminders to support clinical management of patients [8], computer-based skills training [9], and IT-based healthcare professional decision support for clinical decision making [10].

Previous reviews have examined the use of technologies to support healthcare professional practice, such as email to support clinical communication between professionals [11], electronic health information to improve clinical practice (professional behaviors or adherence to clinical practice guidelines) [12], on-screen reminders (such as prompts to conduct a clinical test), and computer-generated reminders delivered on paper [13]. The majority of the preceding literature has tended to focus on effectiveness only, and includes specific interventions, within specific settings, such as electronic health records [14] and computerised provider order entry [15] only. This limits the generalisability of findings to other settings in relation to developing interventions to be delivered at scale.

In addition, it is widely recognized that interventions are most effective when based on behavior change theory and techniques [16, 17]. The use of theory is necessary for explaining and identifying target beliefs involved in clinical practice, and offers a framework for designing and conducting interventions [18, 19]. An important omission from previous reviews is whether technology-based interventions aimed at healthcare professional behaviour change include recognised behaviour change techniques (BCTs), and an understanding of whether such interventions are more or less effective with the inclusion of BCTs. Consequently, there is a need to examine whether interventions with a technological component aimed at changing healthcare professional practice include recognized BCTs, and whether those interventions including BCTs are more effective than interventions without.

There are examples of reviews that focus on implementation of e-health interventions within healthcare settings generally; Ross et al. provide a series of recommendations for implementing e-health interventions across a range of settings [20], however measures of behaviour are not included. Consequently, to build on the previous literature, there is a need to consider the importance of changing healthcare professional practice alongside understanding issues in relation to the implementation of technological interventions. Simply providing healthcare professionals with new technology is unlikely to lead to the transformation in health care that such new technology is proposed to deliver. Specifically, there is a need to conduct an overarching synthesis of diverse technology-based interventions that aim to change healthcare professional behaviour which focuses on three key areas: (1) identifying specific features associated with intervention effectiveness (i.e. what works, for whom interventions for, and under what circumstances interventions work), (2) the BCTs associated with successful interventions, and (3) the barriers and facilitators associated with successful implementation of technology-based interventions. Consequently, there is a need to synthesise and examine intervention characteristics using a methodology suited to identifying important features of effective interventions, and the barriers and facilitators to implementation.

Traditional systematic reviews focus on effectiveness of interventions only. Realist review methods, on the other hand, provide a means of evidence synthesis focused on providing explanations for *how* and *why* interventions may or may not work, and aims to identify features of successful interventions [21]. The advantage of using realist methods over more traditional systematic review methods, is the ability to search for specific explanations regarding implementation of interventions, with no limitations on study design [22–26]. Intervention characteristics (such as study setting, population, and intervention category), as well as the barriers and facilitators of implementation, can be examined using realist review methods to provide a detailed picture of intervention characteristics above and beyond traditional review methods. A realist approach is particularly suited to synthesising evidence about complex interventions [21, 27], including technology-based interventions [28]. This approach determines which interventions work (e.g. computer-based training versus automated reminders), for whom they work (e.g. general practitioners versus nurses), and under what circumstances (e.g. study setting such as primary versus secondary care) they are most effective [29, 30]. This provides rich, detailed and a highly practical understanding of interventions, which is particularly important when planning and implementing interventions on a wider scale [30].

Three specific research questions were addressed:

1. What are the key features of interventions with a technological component that are successful at changing healthcare professional practice?
2. *If* and *how* do such interventions include Behaviour Change Techniques (BCTs) [17] and does the inclusion of BCTs make a difference to practice change?
3. What are the barriers and facilitators to successful implementation of technology-based interventions in practice?

## Methods

A literature review informed by realist review methods was conducted using the five-stage approach [21]: (1) establishing the focus of the review; (2) using a purposive and theoretically driven search strategy and appraisal of literature; (3) searching for multiple types of evidence; (4) using an iterative process throughout; and (5) ensuring the findings explain why (or why not) interventions work and how they work, and provide suggestions for future research and practical application of successful interventions.

### Inclusion criteria

There were no limitations on study design. Interventions targeting any healthcare profession were included. Technology was defined as any aspect of an intervention that involves information technology used as part of patient management strategies (such as computer-generated reminders or alerts).

Studies must have reported: (a) interventions with at least one healthcare professional outcome relating to a change in behavior/practice. For example, changes in professional behavior, action or performance (such as appropriate prescribing or adherence to clinical guidelines); or (b) the practicalities of delivering such interventions using technological supports.

### Search strategy

Systematic searches were conducted in the following electronic databases (up to December 2016): Medline, Embase, Cumulative Index to Nursing and Allied Health Literature (CINAHL), PsycINFO, ISI Web of Science, and Cochrane Library. The reference lists of key systematic review papers were also included in the hand search of all relevant papers. Conference abstracts/reports identified through the database search were only included if they provided sufficient outcome data relating to changes in healthcare professional practice.

A broad search strategy (Additional file 1) was used to capture the widest possible numbers of studies from a range of categories, which included both intervention studies and studies reporting the practicalities of delivering interventions. Medical Subject Headings (MeSH) terms and key words relating to healthcare professional behavior change and technological supports were used.

### Screening

After the initial literature search, two authors (CK and MPT) screened titles and abstracts according to the inclusion criteria. Where abstracts provided insufficient information, full-text review was carried out. Papers meeting the final inclusion criteria were then categorised into two groups; those reporting the results of behavior change interventions aimed at healthcare professionals, and those reporting the practicalities (barriers and facilitators) of delivering such interventions (Additional file 2).

### Data extraction and analysis

Data analysis focused on three phases: (1) a quantitative descriptive analysis to identify and evaluate the *characteristics of interventions*, (2) coding interventions for *recognized behavior change techniques (BCTs)*, and (3) a thematic analysis of the *practicalities of designing and implementing technological interventions.*

1. Characteristics of interventions

Key study characteristics were tabulated using an Excel spreadsheet (including study year, country and healthcare setting). Particular emphasis was given to principles consistent with realist review methodology: the type of intervention used, at whom the intervention was targeted, and the circumstances under which the intervention was described as being effective (target behavior and setting). Specific elements of the intervention were categorised to provide explanations of their effectiveness (a positive change in healthcare professional practice, where *p* < .05, or ineffectiveness, to determine which interventions work) [21]. Study effect sizes were calculated where possible. This included contacting study authors to obtain any missing information. The primary outcome was whether the intervention resulted in a change to healthcare professional clinical practice (both objective and self-reported).

(2) Coding interventions for BCTs

A coding frame was informed by a recognized taxonomy of BCTs [17]. Whilst analysis of behavior change interventions aimed at healthcare professionals has not previously been conducted in the context of technological supports, coding of a similar nature has been conducted in other contexts [31].

Coding was conducted by authors with previous experience of using the BCT taxonomy. One of the study authors (CK) coded the interventions for evidence of BCTs according to the standardised definitions [17], and included both implicit and explicit use of BCTs. A second coder (JH) independently screened a sample selected at random. Disagreements were resolved after discussion, and a third coder (MT) was consulted if agreement could not be reached. An Excel spreadsheet was used to create the coding frame and record intervention descriptions and frequencies of BCTs.

(3) Thematic analysis of the practicalities of designing and implementing technological interventions.

The qualitative software data management tool NVivo was used to sort and categorise the data. Analysis involved coding each study in terms of capturing key ideas and understandings and linking this with the emerging theoretical framework [32]. Thematic analysis was used to provide the best approach to evidence synthesis according to the pre-defined research questions. Findings were summarised under key thematic headings, according to the main findings of each paper, which were used to inform the overall description of the key points [33]. Codes from all identified studies were then compared and cross-referenced, and organised into recurring/higher order themes.

## Results

**Fig. 1:**
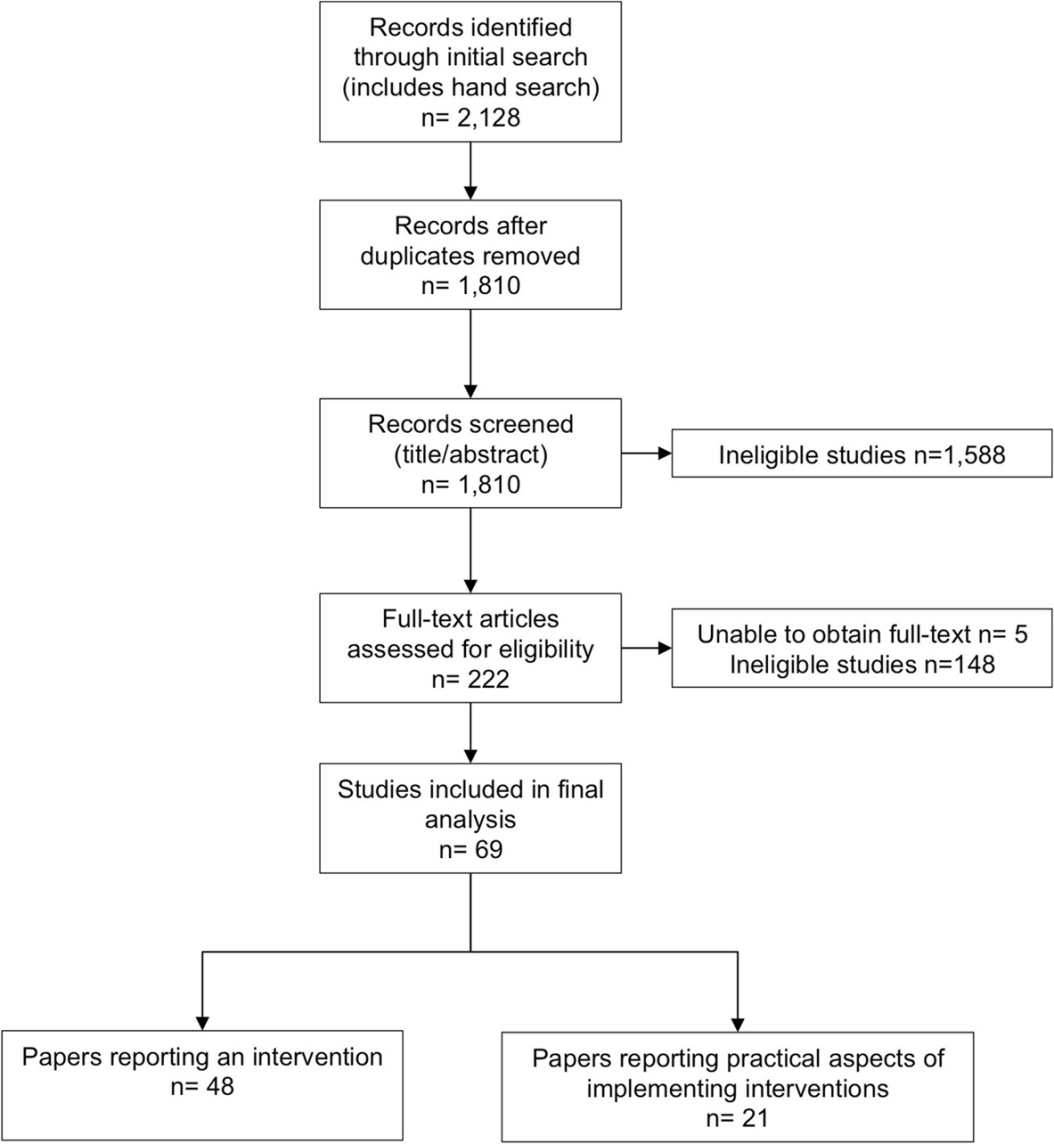
Flow diagram of search strategy

A total of 69 papers were included in the final analysis; 48 studies (of which 27 were randomized controlled trials) were identified in which there was a technological component used to support healthcare professional practice change, and 21 papers reported the practicalities associated with the design and implementation of technology-based interventions (Fig. 1). One paper [34] was included in both parts of the analysis.

### Characteristics of interventions

Forty-eight studies (Table 1) met the inclusion criteria to answer review questions 1 (features of effective behavior change interventions with a technological component), and 2 (if and how behavior change theory was used in the interventions). These were conducted in the USA (*n* = 25), United Kingdom (*n* = 7), The Netherlands (*n* = 4), Australia (n = 4) or elsewhere (*n* = 8). One study did not report the country in which the study was conducted.

**Table 1:** Details of included studies (*n* = 48)

Lead author	Year	Setting	Healthcare professional group	Target behavior	Target behavior (category)	Intervention	Participants randomised	Control group	Significant effect^a^found?	Outcomes	Cohens d	Size
Armstrong [56]	2013	Not reported	Nurse practitioners, physician’s assistant, physician	Initiation of a recommended therapeutic alternative within 90 days of the fax alert for the 13 PDDIs	Prescribing Behaviors	Faxed alerts	N	N	N	Therapy change	na^d^	
Avery [40]	2012	Primary Care	Doctors	Prescribing errors	Prescribing Behaviors	Computer-generated feedback	Y	Y	Y	Prescription problems	0.09^b^	S
										Prescribed B Blocker	0.08^b^	S
										Prescribed an NSAID	0.17^b^	S
Bahrami [9]	2004	Dentist	Dental practitioners	Guideline implementation for the management of impacted and un-erupted third molars in primary dental care	Adherence to clinical patient management guidelines	Computer-based training	Y	Y	N	Guideline implemented	-0.10^b^	S
Beidas [50]	2012	Community Care	Mental health community therapists	Therapist adherence to CBT for child anxiety, skill in CBT for child anxiety, knowledge about CBT for child anxiety, and satisfaction with training.	Adherence to clinical patient management guidelines	Computer-based training	Y	Y	N	Guideline adherence	-0.15^b^	S
Beeckman [57]	2013	Nursing Home	Nurses, nursing assistants, physiotherapists, occupational therapists	Adherence to recommendations to pressure ulcer prevention	Adherence to clinical patient management guidelines	Healthcare professional decision supports	Y	Y	Y	Guideline adherence	1.26^b^	L
Buising [10]	2008	Tertiary Care	Doctors (senior and junior)	Antibiotic prescribing	Prescribing Behaviors	Healthcare professional decision supports	N	N	Y	Concordant therapy	0.76^b^	M
Carton [58]	2002	Hospital	Junior and senior practitioners	Effects of computer-based guidelines on unnecessary medical imaging	Adherence to clinical patient management guidelines	Reminders/ alerts	N	N	Y	Test requests not confirming to guidelines	0.17^b^	S
Cosgrove [59]	2007	Tertiary Care	Clinicians	Inappropriate antimicrobial therapy	Adherence to clinical patient management guidelines	Text message	N	N	N	Guideline adherence	0.19^b^	S
Curtis [60]	2007	Primary Care & Secondary Care	Physicians	To increase bone mineral density (BMD) testing and osteoporosis medication prescribing among patients receiving long term glucocorticoid therapy	Adherence to clinical patient management guidelines & Prescribing Behaviors	Hyperlinks	Y	Y	N		na^d^	
Dimeff [61]	2009	Secondary Care	Mental health treatment providers	Increasing knowledge and self-efficacy and application of course content performance-based role plays	Increasing knowledge, or self-efficacy/confidence	Computer-based training	Y	Y	Y	Knowledge	0.52^c^	M
Dykes [62]	2005	Hospital	Nurses, resident physicians, physical therapists, pharmacist, and dieticians	Adherence to practice guidelines for heart failure	Adherence to clinical patient management guidelines	Healthcare professional decision supports	N	Y	Y		na^d^	
Eccles [63]	2002	Primary Care	GPs and practice nurses	Adherence to the guidelines	Adherence to clinical patient management guidelines	Healthcare professional decision supports	Y	Y	N	Blood pressure recorded	0.00^b^	S
										Exercise recorded or advised	-0.16^b^	S
										Weight recorded or advised	-0.11^b^	S
										Smoking status known	-0.28^b^	S
										Smoking education given	0.00^b^	S
										electrocardiogram recorded	0.00^b^	S
										Exercise electrocardiogra m recorded	0.00^b^	S
										Haemoglobin concentration recorded	0.00^b^	S
										Thyroid function recorded	-0.10^b^	S
										Cholesterol or other lipid concentrations recorded	-0.09^b^	S
										Blood glucose or HBA_1c_ concentrations recorded	0.00^b^	S
Edelman [34]	2014	Primary Care	Physicians	Confidence and knowledge	Increasing knowledge, or self-efficacy/confidence	Healthcare professional decision supports	N	N	Y	Confidence discussing sickle cell disease Confidence conducting follow-up	na^d^	
Fein [41]	2010	Hospital	Clinical staff	Identification of psychiatric problems/ hospital assessments	Increasing screening/testing rates	Healthcare professional decision supports	N	N	Y	Identification of adolescents with psychiatric problems	0.29^b^	S
										ED assessments	0.22^b^	S
Fifield [64]	2010	Hospital	Primary care physicians	Improving both practitioner adherence to National Asthma Education and Prevention Program Guidelines (NAEPP)	Adherence to clinical patient management guidelines	Reminders/ alerts Computer-generated feedback	N	Y	Y	Guideline appropriate prescribing	na^d^	
Filippi [65]	2003	Primary Care	GPs	Increasing the use of antiplatelet drugs for diabetic patients at high-risk to develop future CVD	Prescribing Behaviors	Healthcare professional decision supports	Y	Y	Y	Patients treated	0.36^b^	S
Fortuna [37]	2009	Hospital	Physicians, nurse practitioners and physician assistants	Reducing prescribing of heavily marketed hypnotic medications in ambulatory care settings	Prescribing Behaviors	Reminders/ alerts	Y	Y	Y		na^d^	
Gerber [66]	2013	Primary Care	Paediatricians	Decrease inappropriate antibiotic prescribing for common ARTIs over time by primary care paediatricians	Prescribing Behaviors	Email Feedback	Y	Y	Y		na^d^	
Goetz [4]	2013	Primary Care	Primary care clinicians	Increasing the rate of risk-based and routine HIV diagnostic tests	Increasing screening/testing rates	Reminders/ alerts	Y	Y	Y		na^d^	
Goetz [47]	2008	Primary Care & Secondary Care	Academic and non-academic staff physicians, postgraduate medical trainees and mid-level providers	Increasing the rate of HIV diagnostic testing	Increasing screening/testing rates	Reminders/ alerts Email Feedback	N	Y	Y		na^d^	
Gonzales [67]	2013	Primary Care	Clinicians	Antibiotic treatment of uncomplicated acute bronchitis	Prescribing Behaviors	Healthcare professional decision supports	Y	Y	Y	Unnecessary use of antibiotics	0.46^b^	S
Guldberg [68]	2011	Primary Care	GPs	Initiation of treatment	Clinical intervention/ management	Electronic Feedback System	Y	Y	Y	Oral antidiabetic treatment initiated (1)	0.71^b^	M
										Oral antidiabetic treatment initiated (2)	0.71^b^	M
										Insulin treatment initiated (1)	0.55^b^	M
										Insulin treatment initiated (2)	0.37^b^	S
										Lipid lowering treatment initiated	0.71^b^	M
										Blood-pressure reducing treatment initiated	0.90^b^	L
Gupta [38]	2014	Hospital	Physicians	Appropriate head CT use in patients with mild traumatic brain injury guideline adherence	Adherence to clinical patient management guidelines	Healthcare professional decision supports	N	N	Y		na^d^	
Hibbs [69]	2014	Hospital	Clinicians	Blood transfusion practice of clinicians	Increasing screening/testing rates	Healthcare professional decision supports	N	Y	Y	Transfusion compliance	0.46^b^	S
Hobbs [70]	1996	Primary Care	Primary care practitioners	Prescribing of lipid lowering agents, use of lab tests, and referrals to secondary care for the investigation of hyperlipidaemia	Prescribing Behaviors & Increasing appropriate referrals	Healthcare professional decision supports	Y	Y	N		na^d^	
Hoch [71]	2003	Primary Care	Physicians	Imitating potassium testing	Increasing screening/testing rates	Reminders/ alerts	N	N	Y		na^d^	
Kortteisto [72]	2014	Primary Care	Physicians nurses physiotherapists ward nurses a psychologist	Reminders for best practice guidelines/recommendations	Adherence to clinical patient management guidelines	Healthcare professional decision supports	Y	Y	N		na^d^	
Litvin [73]	2013	Primary Care	Physicians, nurse practitioners, physician’s assistants	Prescribing behavior - antibiotic prescribing for acute respiratory infections	Prescribing Behaviors	Healthcare professional decision supports	N	N	Y		na^d^	
Lobach [74]	1997	Primary Care	Primary care clinicians: family physicians, general internist, nurse practitioners, physician’s assistants, and family medicine residents	Rate of compliance with guideline recommendations for diabetes patient care	Adherence to clinical patient management guidelines	Healthcare professional decision supports	Y	Y	Y	Foot examination	0.62^b^	M
										Complete physical examination	1.07^b^	L
										Chronic glycemia monitoring	0.10^b^	S
										Urine protein determination	2.36^b^	L
										Cholesterol level	0.89^b^	L
										Ophthalmologic examination	1.09^b^	L
										Influenza vaccination	0.18^b^	S
Maiburg [39]	2003	Primary Care	GP trainees	Improving knowledge and practice behavior	Increasing knowledge, or self-efficacy/confidence & Clinical intervention/ management	Computer-based training	N	Y	Y	knowledge test	0.44^c^	S
										correct performance in visit	1.59^c^	L
Malone [75]	2012	Pharmacy	Prescribers	Prevention of serious drug-drug interactions (DDI) prescribing patterns of 25 previously identified clinically important potential DDIs	Prescribing Behaviors	Personal Digital Assistant	N	Y	N	Prescribing at least one DDI	0.20^b^	S
Mayne [76]	2014	Hospital	Physician Nurse practitioner	Captured opportunities for HPV vaccination	Increasing screening/testing rates	Reminder within patient electronic health records	Y	Y	Y		na^d^	
Nilasena [77]	1995	Secondary Care	Physicians	Physician compliance with diabetes preventive care guidelines	Adherence to clinical patient management guidelines	Reminders/ alerts	Y	Y	N		na^d^	
Patkar [78]	2006	Hospital	Breast clinicians (surgeons)	Adherence to guideline recommendations	Adherence to clinical patient management guidelines	Healthcare professional decision supports	Y	Y	Y	adherence to guidelines	1.03^b^	L
Piening [79]	2013	Hospital	Ophthalmologists and hospital pharmacists	Uptake of drug safety information	Adherence to clinical patient management guidelines	Email Feedback	Y	Y	Y	correctly indicated that a serious increase in intra-ocular pressure could be caused by pegaptanib injections	0.86^b^	L
Reeve [80]	2008	Pharmacy	Pharmacists	Frequency of clinical interventions recorded by community pharmacists/to discuss the suitability of aspirin therapy in eligible patients with diabetes	Clinical intervention/ management & Prescribing Behaviors	Healthcare professional decision supports	Y	Y	Y		na^d^	
Ribeiro-Vaz [81]	2012	Hospital	Doctor, nurse, pharmacist	To promote spontaneous adverse drug reaction reporting by healthcare professionals	Prescribing Behaviors	Hyperlinks	N	N	Y		na^d^	
Rocha [82]	2001	Tertiary care	Clinicians - staff physicians, physician assistants, nurse practitioners	Practice patterns and consequently improve the detection and management of nosocomial infections.	Clinical intervention/ management	Reminders/ alerts	N	N	N	Patient management recommendations followed		
Ruland [83]	2002	Hospital	Nurses	Clinicians eliciting and integrating patients’ preferences into patient care	Clinical intervention/ management	Diagnostic/ risk assessment tool	N	Y	Y	congruence between patient preferences and nurse care priorities	0.67^c^	M
Schwarz [84]	2012	Primary Care	Primary care providers	Provision of family planning services when prescribing potentially teratogenic medications	Clinical intervention/ management	Healthcare professional decision supports	Y	Y	Y	discussion of risk of medication use	0.70^c^	M
Sharifi [85]	2014	Primary Care	Physicians	Tobacco smoke exposure management and quit-line referrals	Increasing appropriate referrals	Reminder within patient electronic health records	N	N	Y	counselling for positive screen	1.36^b^	L
Strayer [86]	2013	Primary Care	Physicians	Smoking cessation counselling behaviors, knowledge and comfort/self-efficacy	Increasing knowledge, or self-efficacy/confidence & Clinical intervention/ management	Personal Digital Assistant	N	N	Y		na^d^	
Strom [87]	2010	Hospital	Resident physicians and nurse practitioners	Changing prescribing reduce concomitant orders for warfarin and trimethoprim-sulfamethoxazole,	Prescribing Behaviors	Reminders/ alerts	Y	Y	N		na^e^	
Tang [88]	1999	Secondary Care	Clinicians	Influenza vaccination by clinicians Compliance with the guideline: was defined as documentation that a clinician ordered the vaccine, counselled the patient about the vaccine, offered the vaccine to a patient who declined it, or verified that the patient had received the vaccine elsewhere	Adherence to clinical patient management guidelines	Healthcare professional decision supports	N	N	Y	compliance with guidelines	0.88^b^	L
Tierney [48]	2003	Primary Care	Physicians and pharmacists	Management of heart disease adherence with care suggestions	Adherence to clinical patient management guidelines	Healthcare professional decision supports	Y	Y	N	Compliance with guidelines	0.04^b^	S
Vagholkar [89]	2014	Primary Care	Family physicians	Prescribing - prescription of antihypertensive and lipid-lowering medication.	Prescribing Behaviors	Diagnostic/ risk assessment tool	Y	Y	N	Prescribing of antihypertensive Prescribing of lipid-lowering medication	-0.21^b^	S
van Wyk [8]	2008	Primary Care	GPs	Screening and treatment for dyslipidaemia	Increasing screening/testing rates	Reminders/ alerts	Y	Y	Y	patients screened	0.93^b^	L
										patient treated	0.68^b^	M
Walker [90]	2010	Primary Care	GPs	Increasing opportunistic chlamydia testing	Increasing screening/testing rates	Reminders/ alerts	Y	Y	N	Testing rates	-0.09^b^	S

^a^A significant change in healthcare professional practice, where *p* < .05

^b^Calculated according to Lipsey and Wilson [91] using n in control/intervention conditions based on whether intervention was successful/unsuccessful (2 X 2 frequency table)

^c^Calculated according to Lipsey and Wilson [91] using means (SDs) and sample sizes

^d^Insufficient data to calculate effect size

^e^Unable to calculate due to incomplete study

### Types of intervention

Results are presented in Table 2. The use of healthcare professional decision supports, defined as a decision support system providing healthcare professionals with knowledge and/or person-specific information to assist with patient management [35], was the most commonly used technological intervention (*n* = 19 studies); 15 of the 19 (79%) interventions were effective. We were able to extract effect sizes for 12 studies relating to 29 outcomes (small; *n* = 19, medium; n=; 3, large; *n* = 7) according to definitions provided by Cohen [36].

**Table 2:** Details of success of interventions based on type of intervention and target behavior

Domain	Number of interventions in each category	Number of effective interventions^a^	(%) of effective studies
Intervention type			
Computer-generated feedback	1	1	100
Email feedback	3	3	100
Electronic feedback system	1	1	100
Computer-based training	4	2	50
Reminder system within patient electronic health records	2	2	100
Healthcare professional decision support	19	15	79
Hyperlinks	2	1	50
Reminders/alerts	11	7	64
Personal digital assistant	2	1	50
Diagnostic/risk assessment tool	2	1	33
Faxed alerts	1	0	0
Text message	1	0	0
Target behavior			
Adherence to clinical patient management guidelines	17	10	59
Prescribing behaviors	15	9	60
Increasing screening/testing rates	8	7	88
Clinical intervention/management	6	5	83
Increasing knowledge, or self-efficacy/confidence	4	4	100
Increasing appropriate referrals	2	1	50

^a^A statistically significant change in healthcare professional practice, as described by the authors of each study included in this review

The second most commonly used intervention group was reminders and alerts (*n* = 11 studies), and this also had the second highest percentage of effective interventions (7 of 11 effective; 64%). We were able to extract effect sizes for 3 studies relating to 4 outcomes (small; *n* = 2, medium; *n* = 1, large; *n* = 1).

There were several groups of less frequently used interventions, but that were shown to be effective. One study examined computer-generated feedback, showing positive effects. We were able to extract effect sizes relating to three outcomes (small; *n* = 3). Relating to use of email, 3 of 3 studies showed positive effects. Effect size was calculated for one study relating to one outcome (large; *n* = 1). In the category electronic feedback system, one study showed positive effects, with an effect size relating to six outcomes (small; n = 1, medium; *n* = 4, large; *n* = 1).

Due to the heterogeneity of the studies it was not appropriate to compute summary statistics. In addition due to the varied reporting of study results, we were only able to calculate effect sizes for a sub-sample of papers (*n* = 27), of which there was considerable variation in the size of the effect of reported outcomes (small; *n* = 31, medium; *n* = 10, large; *n* = 12). A forest plot illustrating the range of effect sizes for each outcome of interest is presented in Additional file 3.

### Setting of intervention

Whilst the most common intervention setting for technological interventions was primary care (*n* = 23; 48%), studies conducted in hospitals (*n* = 14; 28%) had a higher success rate (12 of 14 described as effective; 86%). Other less frequent settings included interventions conducted within both primary and secondary care (1 of 2 effective; 50%).

### Target healthcare professional for intervention

Half of the interventions were targeted at General Practitioners (*n* = 24; 50%), with this group also having the highest success rate (18 of 24 studies [75%] resulting in professional behavior change). The second largest group were interventions targeted at two or more types of healthcare professional (*n* = 16; 33%), over half of which resulted in practice change (10 of 19; 61%). There were several other groups of less frequently targeted healthcare professionals among whom technology-based interventions had been tested. These included interventions targeted at mental health therapists (2 of 2 effective; 100%) and pharmacists (1 of 2 effective; 50%).

### Target behavior of intervention

Interventions according to target behavior are presented in Table 2. The most common behavior targeted by technological interventions was adherence to clinical guidelines for patient management (*n* = 17; 35%), over half of which were effective in changing practice (10 of 17 studies; 61%).

The second most commonly targeted behavior was prescribing behaviors (*n* = 15; 31%); half of the studies resulted in practice change (9 of the 15 studies; 60%). There were other less frequently targeted behaviors that demonstrated high success rates, including studies targeting increased knowledge or self-efficacy/confidence (4 out of 4 [100%] effective), increasing screening/testing rates (7 out of 8 [88%] effective), and clinical intervention/management (5 out of 6 [83%] effective) all were described as showing positive effects.

### Coding interventions for specific BCTs

**Fig. 2:**
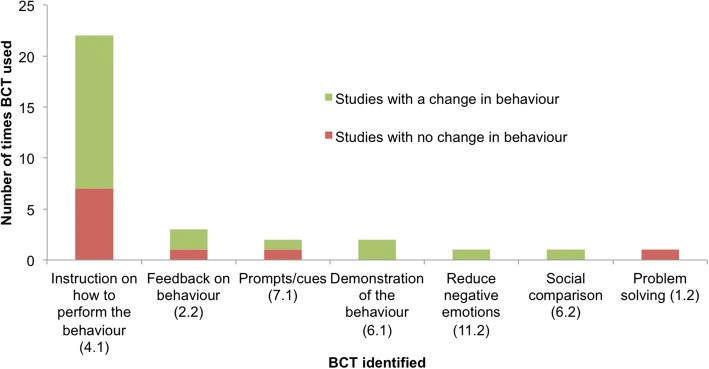
Behavior change techniques identified across 26 studies according to Michie et al. [17]

Of the 48 studies included in the final analysis, 26 (54%) contained evidence of BCTs relating to use of technology or the target behavior (Fig. 2). Seven different BCTs were identified across the 26 studies. The BCT code according to Michie et al. [17] is presented in parentheses, followed by the number of studies using each technique. The most commonly used BCT was *instruction on how to perform the behavior* (BCTTv1 4.1; *n* = 22). This technique was mostly used in the context of healthcare professional decision support interventions (*n* = 9), and reminders and alerts (*n* = 9). Other techniques included *feedback on behavior* (BCTTv1 2.2; *n* = 3), *prompts/cues* (BCTTv1 7.1; *n* = 2), *demonstration of the behavior* (BCTTv1 6.1; *n* = 2), *reducing negative emotions* (BCTTv1 11.2; *n* = 1), *social comparison* (BCTTv1 6.2; *n* = 1), and *problem solving* (BCTTv1 1.2; *n* = 1).

Of the 26 studies containing evidence of BCTs, 16 studies (62%) resulted in practice change (Fig. 2). Of these, five different BCTs were used across the 16 studies. The largest group was *instruction on how to perform the behavior* (BCTTv1 4.1; *n* = 15), such as instructing healthcare professionals which medicines to prescribe [37] or requesting an appropriate clinical test [38]. This second largest group was *demonstration of the behavior* (BCTTv1 6.1; *n* = 2), such as demonstrating effective clinician practice [39].

### Thematic analysis

To answer review question 3 (What are the barriers and facilitators to successful implementation of such technology-based interventions in practice?), a thematic analysis was conducted to address the practicalities of implementing technology in practice. Characteristics of the 21 qualitative (*n* = 19) and quantitative (*n* = 2) studies are presented in Table 3.

**Table 3:** Details of practicalities papers (*n* = 21)

Lead author	Year	Country	Data collection method
Ackerman [43]	2013	USA	Qualitative structured telephone surveys
Barnett [92]	2015	UK	Think-aloud and semi-structured interviews
Bokhour [93]	2015	USA	Qualitative semi-structured interviews
Burns [94]	2007	Australia	Semi-structured interviews
Doerr [95]	2014	USA	Semi-structured interviews
Dowding [96]	2009	UK	In-depth semi-structured interviews
Dryden [97]	2012	USA	Qualitative, in-depth semi-structured telephone interviews
Edelman [34]	2014	USA	Semi-structured interviews and quantitative survey data
Guldberg [98]	2010	Denmark	Group and individual semi-structured interviews
Hains [99]	2009	Australia	Semi-structured interviews
Litvin [100]	2012	USA	Semi-structured group interviews
Maguire [101]	2008	UK	Questionnaires and semi-structured interviews
Mandt [102]	2010	Norway	Focus groups
Patterson [42]	2004	USA	Semi-structured interviews
Power [103]	2014	Canada	Surveys
Randell [104]	2010	UK	In-depth semi-structured interviews
Rousseau [105]	2003	UK	Semi-structured interviews
Saleem [106]	2005	USA	Qualitative field observations
Vishwanath [107]	2009	USA	Surveys
Weir [108]	2011	USA	Formative interviews
Zhu [109]	2015	USA	Qualitative Survey

**Fig. 3:**
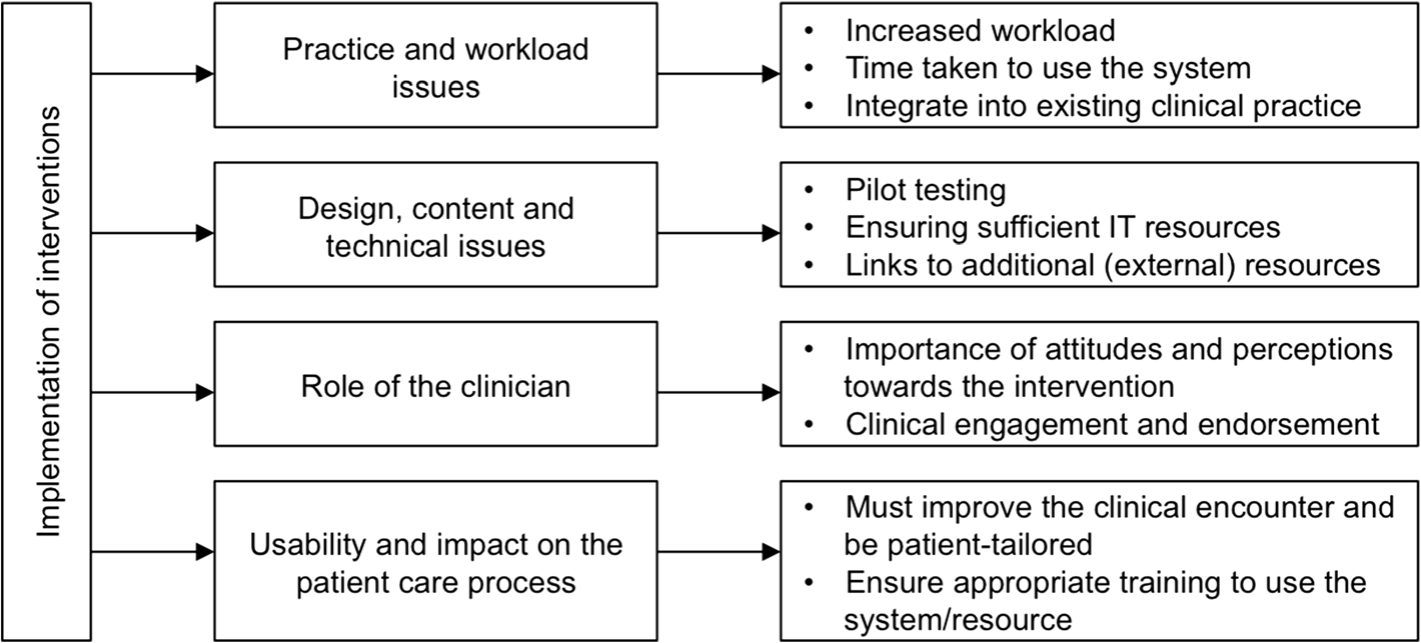
Barriers and facilitators of implementing technological support interventions aimed at supporting

Themes identified address the barriers and facilitators of implementing and delivering technology-based interventions aimed at supporting professional practice change. Four major themes were identified (summarised in Fig. 3): (1) *Practice and workload issues,* (2) *Design, content and technical issues,* (3) *Role of the healthcare professional,* and (4) *Usability and impact on the patient care process.* The numbers of papers reporting each finding are reported in parentheses, out of a possible 21 papers.

1. Practice and workload issues

A number of contextual features (the setting in which the intervention was delivered) were highlighted; practice and workload considerations were perceived as important in the implementation of technology-based interventions. Increased workload was an issue as a result of using technology as part of everyday practice, which may disrupt the workflow of healthcare professionals (*n* = 4; 19%). Time taken to use the system was cited as a barrier to likelihood of healthcare professionals using technology (*n* = 5; 24%), suggesting a need for a more user-friendly design of technology-based interventions. However, technology was seen as a way of improving communication between healthcare professionals (*n* = 3; 14%) and improving the delivery of healthcare in practice (*n* = 3; 14%).

A key factor for the successful implementation of technology into the healthcare environment was whether the intervention met the practice/organizational goals and objectives (*n* = 11; 52%). More specifically, whether the addition of technology met current practice initiatives and could be easily integrated into existing clinical practice, and targeted organizational incentives including patient management approaches and financial incentives.

(2) Design, content and technical issues,

Features of the technology itself were also highlighted. Studies emphasised the importance of pilot testing before wide-scale usage and in particular the need to take an iterative modification approach, such as customising tools to the needs of the staff (*n* = 5; 24%). Piloting may identify important technical issues acting as barriers to usage, such as insufficient access to IT resources, software updates and limitations in computer performance (*n* = 9; 43%). Where interventions included patient management guidelines, the need for consistency and reliability was highlighted. Links to external resources such as forums, risk assessment tools or patient information sources, must be used appropriately and in a way to improve the delivery of patient care (*n* = 8; 38%). Guidelines in particular must be relevant to patient management (*n* = 4; 19%).

(3) Usability and benefit for patient care.

Additional important features of the technology included accessibility to important information relevant to the clinical encounter for example medication information, hence making it an important educational resource (*n* = 4; 19%). Technology was also seen as a way of improving other aspects of the clinical encounter such as medication reviews or stimulating provider-patient discussions (*n* = 5; 24%).

An important feature was the ease of use of the technology (*n* = 9; 43%); barriers included system navigation and poor interface issues. A major factor in the uptake and acceptability of technology-based interventions was appropriate training and IT skills (*n* = 18; 86%). Specific considerations include implementing an initial learning/familiarisation period to use the system and providing technical training for users.

(4) Role of the healthcare professional

Technology-based interventions increase healthcare professional confidence in decision making in situations of uncertainty around patient management (*n* = 3; 14%). Attitudes and perceptions of healthcare professionals towards technologies were seen as important in terms of its usage; positive attitudes were more commonly associated with uptake (*n* = 6; 29%). Two studies emphasised the importance of senior professionals endorsing and driving the use of technology as being key to its success. In particular, healthcare professional engagement with technologies was reported as being key to its implementation, such as assigning one or more groups of healthcare professionals with sole responsibility of using the system, such as practice nurses (*n* = 6; 29%).

## Discussion

This review has identified key features of successful interventions with a technological component aimed to improve healthcare professional practice. Results provide insights into the characteristics of successful interventions and provide recommendations for the design and implementation of technologies based on the barriers and facilitators identified.

A summary of the key findings from the present review is presented in Table 4, which outlines successful intervention features and components, effective BCTs used in interventions, and the barriers and facilitators in relation to implementing interventions. The most successful technological intervention was healthcare professional decision support, suggesting this may have an important role to play in clinical practice. The most common intervention *setting* was within primary care; however more practice change occurred in hospitals. This suggests two areas for future research. First, it is necessary to understand how the hospital setting, a key place in which deliver behavior change interventions aimed at supporting healthcare professional practice, such as prescribing practices [40] or screening of health conditions [41] can be utilised to facilitate delivery of technological interventions. Second, research is needed to find ways of overcoming the barriers that exist within primary care settings, particularly those identified by our thematic analysis. For example, organizational/structural and logistical barriers such as workload and time pressures are often cited as challenges in primary care settings [42, 43], which may consequently influence the effectiveness of interventions. Our review also suggests financial incentives may be a way of engaging healthcare professionals with interventions. Whilst recent evidence suggests financial incentives may not influence long-term practice habits [44], our findings suggest this may be used to engage clinicians in technology-based interventions and therefore focusing on improving uptake.

**Table 4:** Summary of findings of the important factors of implementation of technological interventions aimed at improving professional practice

Construct	Topic	Specific features / recommendations	References	Barrier / facilitator
What works	Type of intervention	Healthcare professional decision support	[10, 34, 38, 41, 57, 62, 65, 67, 69, 73, 74, 78, 80, 84, 88]	
		Reminders and alerts	[4, 8, 37, 47, 58, 64, 71]	
	BCTs	Instruction on how to perform the behaviour (BCTTv1 4.1)	[8, 10, 37–40, 47, 50, 57, 58, 64, 74, 78, 86]	
For whom interventions work for	Target healthcare professional behaviour	Adherence to clinical guidelines for patient management	[38, 57, 58, 62, 64, 74, 78, 79, 88]	
		Prescribing behaviours	[10, 37, 40, 65–67, 73, 80, 81]	
		Increasing knowledge or self-efficacy / confidence	[34, 39, 86, 61]	
		Increasing screening / testing rates	[4, 8, 41, 47, 69, 71, 76]	
		Clinical intervention / management	[68, 80, 83, 84, 86]	
	Target healthcare professional	GPs	[4, 8, 10, 34, 37, 39, 40, 47, 64–68, 71, 73, 74, 84–86]	
		Multiple healthcare professionals (more than two different types of healthcare professional)	[37, 41, 47, 57, 62, 73, 74, 76, 79, 81]	
Under what circumstances	Role of the healthcare professional	Increases confidence in decision making	[43, 96, 103]	Facilitator
		Attitudes and perceptions towards technology important in terms of uptake and usage	[43, 99–102, 107]	Facilitator
		Importance of endorsement from senior peers	[99, 104]	Facilitator
		Engagement important factor for implementation	[95, 100, 104]	Facilitator
		Assigning responsibility to using the system	[34, 100, 106]	Facilitator
				Facilitator
	Design, content and technical issues	Pilot testing - iterative modification to meet staff needs	[100, 101,104, 106, 108]	Facilitator
		Insufficient access to IT resources	[34, 92, 99, 104]	Barrier
		Physical location of computer	[94, 100, 106]	Barrier
		Technical issues such as computer performance and software updates	[94, 95, 98, 100, 102, 104, 105, 108]	Barrier
		Links to external patient information resources important	[92, 95, 99]	Facilitator
		Links to patient guidelines must be readily available, consistent and relevant	[43, 97–100]	Facilitator
	Usability and benefit for patient care	Provides access to important information relevant to the clinical encounter	[98, 99, 103, 109]	Facilitator
		Technology / interface must not be difficult to use	[43, 93, 96, 97, 99, 104–106, 109]	Barrier
		Technical training for staff	[34, 42, 99–101, 103–105, 107, 108]	Facilitator
		Importance of a learning period / time for familiarisation of the technology	[95–98, 108]	Facilitator
		Considers complexities of individual patients (for example patients with specific conditions, or comorbidities)	[42, 43, 97, 105]	Facilitator
		Helps facilitate discussions with patients	[100, 102]	Facilitator
	Practice and workload issues	Use of technology increases workload and may cause disruption	[34, 42, 97, 106]	Barrier
		Time taken to use the system / requirement of additional staff members	[34, 93, 98–100]	Barrier
		Improves communication between healthcare professionals	[92, 98, 103]	Facilitator
		Must be easily integrated into day-to-day workload	[34, 43, 97, 106]	Facilitator
		Technology aligns with current practice initiatives, and wider organisational context	[43, 92, 95, 98, 105, 108]	Facilitator

The barriers and facilitators identified in this review are consistent with theoretical approaches to understanding implementation of interventions. Normalisation Process Theory [45, 46] can be used to understand how technological interventions become embedded in clinical practice. BCTs can be applied to demonstrate how interventions can be delivered in practice to facilitate implementation of technological interventions. Thus, four key recommendations can be made. First, it is necessary to understand how healthcare professionals make sense of the intervention in question. Consequently, technological interventions must have a clear function and meet organizational initiatives (*coherence* domain; e.g. *instruction on how to perform the behaviour* [BCTTv1 4.1]). Second, healthcare professionals must be actively engaged with technological interventions, which must be endorsed by key professionals within organisations, (*cognitive participation* domain; e.g. *social support* [BCTTv1 3.1]). Third, interventions must be easily integrated into clinical practice by: (a) complementing existing workloads of healthcare professionals; and (b) considering the diversity in terms of the setting in which they are delivered, the recipient of the intervention, and the target behaviour (*collective action* domain; e.g. *action planning* [BCTTv1 1.4]). Fourth, ensure that interventions are appraised by the recipients as having a benefit in terms of improving the patient encounter (*reflexive monitoring* domain; e.g. *self-monitoring of outcome(s) of behaviour* [BCTTv1 2.4]).

Our review shows that General Practitioners (GPs) are the most commonly targeted *healthcare professional* for technology-based interventions, and such interventions demonstrate the highest proportion of success in achieving behavior change. The role of the GP may be particularly important in understanding how technological approaches can be used to support professional practice. Of the 24 studies aimed at GPs, eight studies used computerised decision support and a further eight used reminders and alerts. The second largest group involved targeting multiple healthcare professionals, however only half of the studies resulted in behavior change. This is particularly important as part of the healthcare professional role involves referral and signposting to other healthcare professionals, where appropriate, and is recognized in primary care training strategies [5, 6]. One possible application of technological support, suggested by our thematic analysis, is to use technology to improve the communication between multiple healthcare professionals regarding patient management [11].

### Use of behaviour change techniques in interventions

Use of recognized behavior change techniques [17] was identified in a number of studies. The most commonly used BCT was *instruction on how to perform the behavior* in the context of instructions from decision support systems, reminders and alerts. This technique may be particularly important for supporting healthcare professional clinical practice in the context of a technological intervention, which often involves tasks related to clinical decision making, such as making referrals and conducting health checks [5, 38, 47, 48].

When using BCTs, there were more studies resulting in healthcare professional behavior change than those showing no change. Given that the BCT framework is still in its infancy, interventions must apply the techniques to important areas of clinical practice (such as increasing appropriate screening and more appropriate medicine prescribing practices). The use of theory offers valuable insights both in terms of understanding and supporting practice change [19, 49], and as a framework to guide interventions. Our review has provided encouraging findings supporting the use of BCTs as part of technology-based interventions supporting healthcare professional practice change. Findings suggest that BCTs can be effective across a range of diverse interventions, target behaviours, and healthcare professionals groups. The BCT *instruction on how to perform the behaviour*, effective in 15 studies, was implemented across prescribing behaviours (e.g.), adherence to patient management guidelines (e.g. [50]), and increasing screening rates (e.g.), and found to be effective when delivered to doctors, therapists [50], nurses, and surgeons. Identifying effective BCTs in this way allows the opportunity to deliver interventions aiming to change healthcare professional practice shown to be effective across a range of diverse contexts. Given that 22 of the 48 studies included in this review did not contain any evidence of BCTs, there is considerable scope for future research to develop interventions that include BCTs. This may involve targeting known psychological constructs involved in behavior change, using established as well as emerging frameworks specifically relating to implementation of interventions [51–53].

### Strengths and limitations of this review

Although there are a number of systematic reviews [11–15, 54] that examine the effectiveness of individual types of technology-based interventions aimed at healthcare professionals, this is the first attempt to synthesise evidence across all interventions that include a technological component, and the factors involved in implementation of interventions. We have synthesised the findings from across a diverse range of intervention contexts and settings, and presented a series of barriers and facilitators that are shared across healthcare behaviours and diverse professional groups. The advantage of this approach is this provides a series of recommendations concerning implementation of interventions, and an opportunity for behaviour change interventions to be delivered at scale, targeting multiple healthcare professional groups working in different healthcare settings. This is also an attempt to move beyond the most commonly researched interventions and provide a wider understanding of both intervention function and content. The current review extends the findings of previous reviews by: (1) identifying specific features associated with successful interventions, (2) highlighting opportunities to improve the design of technologies by incorporating known BCTs; and (3) identifying the barriers and facilitators to successful implementation. Future reviews would benefit from including an analysis of patient outcomes, particularly whether changes in healthcare professional practice as a consequence of implementing technological interventions translates into positive patient outcomes.

The realist method of literature review was chosen to guide the present review in order to understand a large and complex literature, with the qualitative findings advancing our quantitative findings by providing an explanatory framework about why and how technological interventions work. This level of detail would not have been possible to identify using the Cochrane style of systematic review methods. Whilst we sought to extract effect sizes for included studies, due to the varied reporting of study results, and in the absence of the relevant statistical information such as *p* values and sample sizes, it was only possible to calculate effect sizes for a small number of papers. Further, due to the range of outcomes obtained, and often multiple outcomes from individual studies, a direct comparison between groups was not possible.

## Conclusions

Technological approaches to improving healthcare professional practice provide opportunities to address challenges in multiple areas of clinical practice [55]. Healthcare professional decision support interventions, when developed using recognized psychological theory such as providing instruction on how to implement interventions, show considerable promise. Interventions must also address known organizational challenges associated with specific settings, as well as focusing on efficiency and user-friendly design content, whilst ensuring interventions complement the day-to-day workload and current knowledge and skillset of the target healthcare professional. Understanding the most important contextual features, and how to apply theoretical insights known to change behavior can all contribute to the design and successful implementation of technologies aiming to directly influence the clinical management of patients.

## Additional files

Additional file 1: Search strategy. (DOCX 15 kb)

Additional file 2: Flow diagram of abstract screening process for each analysis component. (DOCX 22 kb)

Additional file 3: Forest plot displaying Cohen’s d and confidence intervals for study outcomes. (DOCX 34 kb)

## Abbreviations

BCT: Behaviour change technique
BCTTv1: Behaviour change technique taxonomy version 1
CINAHL: Cumulative index to nursing and allied health literature
GP: General practitioner
IT: Information technology
MeSH: Medical subject heading
