# Supplementary material for: What maximizes the effectiveness and implementation of technology-based interventions to support healthcare professional practice? A systematic literature review
Source: BMC Med Inform Decis Mak. 2018 Nov 7;18:93. doi: 10.1186/s12911-018-0661-3 (PMC6223001; doi:10.1186/s12911-018-0661-3)
Supplement: Supplementary file 1 — Search strategy. (DOCX 15 kb) [file 12911_2018_661_MOESM1_ESM.docx]

**Additional File 1 – Search strategy**

Database searches were based on subject headings and related free-text terms. The main search terms were broad and covered participants, intervention type, and outcomes. The following search terms were used, adapted for each database:

1. healthcare professional$.mp. [mp=ti, ot, ab, sh, hw, kw, nm, kf, px, rx, an, ui, tc, id, tm, tn, dm, mf, dv]

2. health professional$.mp. [mp=ti, ot, ab, sh, hw, kw, nm, kf, px, rx, an, ui, tc, id, tm, tn, dm, mf, dv]

3. practitioner$.mp. [mp=ti, ot, ab, sh, hw, kw, nm, kf, px, rx, an, ui, tc, id, tm, tn, dm, mf, dv]

4. clinician$.mp. [mp=ti, ot, ab, sh, hw, kw, nm, kf, px, rx, an, ui, tc, id, tm, tn, dm, mf, dv]

5. 1 or 2 or 3 or 4

6. behaviour change.mp. [mp=ti, ot, ab, sh, hw, kw, nm, kf, px, rx, an, ui, tc, id, tm, tn, dm, mf, dv]

7. behaviour.mp. [mp=ti, ot, ab, sh, hw, kw, nm, kf, px, rx, an, ui, tc, id, tm, tn, dm, mf, dv]

8. behavior change.mp. [mp=ti, ot, ab, sh, hw, kw, nm, kf, px, rx, an, ui, tc, id, tm, tn, dm, mf, dv]

9. behavior.mp. [mp=ti, ot, ab, sh, hw, kw, nm, kf, px, rx, an, ui, tc, id, tm, tn, dm, mf, dv]

10. practice.mp. [mp=ti, ot, ab, sh, hw, kw, nm, kf, px, rx, an, ui, tc, id, tm, tn, dm, mf, dv]

11. 6 or 7 or 8 or 9 or 10

12. computer-assisted.mp. [mp=ti, ot, ab, sh, hw, kw, nm, kf, px, rx, an, ui, tc, id, tm, tn, dm, mf, dv]

13. technology.mp. [mp=ti, ot, ab, sh, hw, kw, nm, kf, px, rx, an, ui, tc, id, tm, tn, dm, mf, dv]

14. electronic.mp. [mp=ti, ot, ab, sh, hw, kw, nm, kf, px, rx, an, ui, tc, id, tm, tn, dm, mf, dv]

15. computerized.mp. [mp=ti, ot, ab, sh, hw, kw, nm, kf, px, rx, an, ui, tc, id, tm, tn, dm, mf, dv]

16. computerised.mp. [mp=ti, ot, ab, sh, hw, kw, nm, kf, px, rx, an, ui, tc, id, tm, tn, dm, mf, dv]

17. informatics.mp. [mp=ti, ot, ab, sh, hw, kw, nm, kf, px, rx, an, ui, tc, id, tm, tn, dm, mf, dv]

18. 12 or 13 or 14 or 15 or 16 or 17

19. intervention.mp. [mp=ti, ot, ab, sh, hw, kw, nm, kf, px, rx, an, ui, tc, id, tm, tn, dm, mf, dv]

20. trial.mp. [mp=ti, ot, ab, sh, hw, kw, nm, kf, px, rx, an, ui, tc, id, tm, tn, dm, mf, dv]

21. randomised controlled trial.mp. [mp=ti, ot, ab, sh, hw, kw, nm, kf, px, rx, an, ui, tc, id, tm, tn, dm, mf, dv]

22. randomized controlled trial.mp. [mp=ti, ot, ab, sh, hw, kw, nm, kf, px, rx, an, ui, tc, id, tm, tn, dm, mf, dv]

23. controlled trial.mp. [mp=ti, ot, ab, sh, hw, kw, nm, kf, px, rx, an, ui, tc, id, tm, tn, dm, mf, dv]

24. 19 or 20 or 21 or 22 or 23

25. 5 and 11 and 18 and 24
