# Supplementary material for: What maximizes the effectiveness and implementation of technology-based interventions to support healthcare professional practice? A systematic literature review
Source: BMC Med Inform Decis Mak. 2018 Nov 7;18:93. doi: 10.1186/s12911-018-0661-3 (PMC6223001; doi:10.1186/s12911-018-0661-3)
Supplement: Supplementary file 2 — Flow diagram of abstract screening process for each analysis component. (DOCX 22 kb) [file 12911_2018_661_MOESM2_ESM.docx]

**Additional File 2.** Flow diagram of abstract screening process for each analysis component

Papers meeting primary inclusion criteria

Papers reporting a healthcare professional behavioural outcome

Papers reporting the practicalities of delivering interventions

Data Extraction

Evidence synthesis
