# Supplementary material for: What maximizes the effectiveness and implementation of technology-based interventions to support healthcare professional practice? A systematic literature review
Source: BMC Med Inform Decis Mak. 2018 Nov 7;18:93. doi: 10.1186/s12911-018-0661-3 (PMC6223001; doi:10.1186/s12911-018-0661-3)
Supplement: Supplementary file 3 — Forest plot displaying Cohen’s d and confidence intervals for study outcomes. (DOCX 34 kb) [file 12911_2018_661_MOESM3_ESM.docx]

**Additional File 3.** Forest plot displaying Cohen’s d and confidence intervals for study outcomes

CI upper

0.27

0.14

0.25

0.68

0.11

1.73

1.11

0.11

0.49

0.91

1.11

-0.02

-0.01

-0.18

0.23

0.13

0.29

0.10

0.01

0.00

0.10

0.41

0.37

0.42

-0.36

0.89

0.99

0.69

0.51

1.00

1.28

0.83

1.16

1.38

0.34

2.86

1.20

1.56

0.43

1.02

2.25

0.37

1.38

1.64

1.06

1.44

1.74

1.20

0.19

-0.05

1.04

0.78

-0.04

CI lower

-0.10

0.02

0.09

-0.89

-0.41

0.79

0.40

0.11

-0.10

0.12

-0.11

-0.31

-0.21

-0.39

-0.23

-0.13

-0.29

-0.10

-0.21

-0.18

-0.10

0.18

0.07

0.30

-0.59

0.52

0.44

0.41

0.23

0.41

0.51

0.08

0.08

0.76

-0.14

1.86

0.58

0.63

-0.08

-0.15

0.93

0.03

0.68

0.08

0.27

-0.04

0.98

0.57

-0.10

-0.38

0.82

0.58

-0.13

ES

0.09

0.08

0.17

-0.10

-0.15

1.26

0.76

0.17

0.19

0.52

0.00

-0.16

-0.11

-0.28

0.00

0.00

0.00

0.00

-0.10

-0.09

0.00

0.29

0.22

0.36

0.46

0.71

0.71

0.55

0.37

0.71

0.90

0.46

0.62

1.07

0.10

2.36

0.89

1.09

0.18

0.44

1.59

0.20

1.03

0.86

0.67

0.70

1.36

0.88

0.04

-0.21

0.93

0.68

-0.09
